# Understanding the value of adhering to or adapting evidence-based interventions: a study protocol of a discrete choice experiment

**DOI:** 10.1186/s43058-021-00187-w

**Published:** 2021-08-11

**Authors:** Ulrica von Thiele Schwarz, Aaron R. Lyon, Kristoffer Pettersson, Fabrizia Giannotta, Pernilla Liedgren, Henna Hasson

**Affiliations:** 1 School of Health, Care and Social Welfare, Mälardalen University, Box 883, Västerås, Sweden; 2 Procome, Medical Management Centre, Karolinska Institutet, Stockholm, Sweden; 3 Department of Psychiatry and Behavioral Sciences, University of Washington, Seattle, Washington USA; 4 Unit for Implementation and Evaluation, Center for Epidemiology and Community Medicine, Region Stockholm, Stockholm, Sweden

**Keywords:** Professionals, Adaptation, Adherence, Fidelity–adaptation dilemma, Evidence-based interventions, Decision-making, Parental support, Discrete choice experiment

## Abstract

**Background:**

Whereas the value of an evidence-based intervention (EBI) is often determined by its effect on clinical outcomes, the value of implementing and using EBIs in practice is broader, reflecting qualities such as appropriateness, equity, costs, and impact. Reconciling these value conflicts involves a complicated decision process that has received very limited scholarly attention. Inspired by studies on decision-making, the objective of this project is to explore how practitioners appraise the values of different outcomes and to test how this appraisal influences their decisions surrounding the so-called fidelity–adaptation dilemma. This dilemma is related to the balance between using an EBI as it was designed (to ensure its effectiveness) and making appropriate adaptations (to ensure alignment with constraints and possibilities in the local context).

**Methods:**

This project consists of three sub-studies. The participants will be professionals leading evidence-based parental programs in Sweden and, in Sub-study 1, parents and decision-makers. Sub-study 1 will use sequential focus groups and individual interviews to explore parameters that influence fidelity and adaptation decisions—the dilemmas encountered, available options, how outcomes are valued by practitioners as well as other stakeholders, and value trade-offs. Sub-study 2 is a discrete choice experiment that will test how value appraisals influence decision-making using data from Sub-study 1 as input. Sub-study 3 uses a mixed-method design, with findings from the two preceding sub-studies as input in focus group interviews to investigate how practitioners make sense of findings from optimal decision situations (experiment) and constrained, real-world decision situations.

**Discussion:**

The project will offer unique insights into decision-making processes that influence how EBIs are used in practice. Such knowledge is needed for a more granular understanding of how practitioners manage the fidelity–adaptation dilemma and thus, ultimately, how the value of EBI implementation can be optimized. This study contributes to our knowledge of what happens once EBIs are adopted—that is, the gap between the way in which EBIs are *intended* to be used and the way in which they *are* used in practice.

Contributions to the literature

- The fidelity and adaptation debate has primarily focused on how fidelity and adaptation affect effectiveness, whereas in practice, practitioners need to consider multiple types of outcomes.
- Although there is hardly any implementation without adaptation, little is known about how practitioners make decisions that lead to fidelity or adaptations.
- New insights will be gained regarding how practitioners value different outcomes when using EBIs and how their appraisals impact their adaptation decisions.
- The study will provide insights into how discrete choice experiments can be added to the methodological arsenal of implementation research.

## Background

The implementation of behavioral evidence-based interventions (EBI) inevitably involves decisions that lead either to high *fidelity* or to *adaptations* [1, 2]. On the one hand, there is a need to ensure that EBIs can be delivered as originally designed (i.e., with high fidelity). On the other hand, when EBIs are used in practice, constraints and opportunities in the local context often prompt practitioners to make adaptations (i.e., deliberate actions to change the content or delivery so that it fits the context [2]). This implies that a choice often needs to be made between actions that promote either high fidelity or adaptation of the EBI*.* This decision, sometimes referred to as the “fidelity–adaptation dilemma” [3], is a critical but underexplored concern for implementation research [4]. The way in which people appraise the *value* of different decision outcomes is of particular importance to gain a better understanding of how fidelity–adaptation decisions are made.

### Appraising the value of EBI outcomes

Many studies have examined how fidelity and different types of adaptations [5, 6] affect efficacy and effectiveness [7–9]. Mixed findings suggest that both high fidelity and significant adaptations may improve the effects of an EBI. However, efficacy is only one of many values that an EBI can optimally produce, making value a multicomponent, multilevel construct that represents the combination of benefits [2]. Other potentially relevant values are recognized in service outcomes, such as safety, timeliness, efficiency, cost-effectiveness, equity, and user (patient)-centeredness, as well as implementation outcomes, such as appropriateness, acceptability, and feasibility [10]. In line with this, empirical research supports the appropriateness of considering the value of fidelity and adaptations in relation to outcomes such as sustainability [11–13], reach [14], feasibility [6], equity [15], person-centeredness [16], and cost-effectiveness [17]. Thus, the value of an EBI can be appraised against several different outcomes. The perceived value of achieving a certain outcome can vary, which means that the reconciliation of the fidelity–adaptation dilemma depends on an *appraisal* of the outcome and the perceived likelihood of achieving it. For instance, it has been suggested that for some stakeholders, such as policy makers, cost-effectiveness may be a more important driver of decisions to adopt and use EBIs than efficacy [17].

The appraisal process is complicated by the fact that each decision concerning fidelity and adaptations may affect different outcomes in different ways. For example, a practitioner may omit certain content of an EBI that is perceived as culturally inappropriate to increase acceptability but may, at the same time, decrease the efficacy of the EBI [18, 19]. The preferred solutions to the fidelity–adaptation dilemma vary depending on how important it is to achieve a certain outcome. Thus, the value of an EBI reflects an appraisal of the *configuration* of all potential outcomes across service users, providers, organizations, and systems [2].

This means that decisions about fidelity and adaptations need to be made on the basis of a holistic judgment of how the decision will affect the configuration of outcomes. Thus, individuals take several potential outcomes into account simultaneously [20]. For example, it has been shown that practitioners take patient-related as well as financial and system-related factors into account in clinical decisions [21]. Such holistic judgments can be difficult since the multiple outcomes may not align [22]. This represents a value conflict since a certain choice may improve one outcome at the expense of another. Despite the ubiquity of these dilemmas, there is a surprising lack of discussion in the implementation literature about how different outcomes are valued and how this appraisal drives decisions about fidelity and adaptation.

Intervention and implementation studies have generally focused on the impact of EBIs or their implementation on a small set of outcomes. There is a shortage of theoretically based empirical investigation into how multicomponent value appraisals of outcomes are made and how they influence decisions related to fidelity and adaptation. Studies have shown that practitioners make adaptations for various reasons, such as to satisfy patient preferences [18], to make the EBI culturally appropriate [23], and to retain patients in the program [24]. However, these studies have not explicitly focused on how practitioners value and negotiate different outcomes in their decision-making. Moreover, although some empirical studies have suggested that decision-makers and practitioners (as well as researchers) may favor different outcomes [17, 25], there is a lack of studies directly contrasting different stakeholder perspectives on valued outcomes.

Thus, although some studies have indicated the type of outcomes that practitioners value (e.g., meeting patient needs [18], appropriateness [23], and patient retention [24]), they have not addressed the relationship between options and outcomes, the way in which outcomes are combined to make an option attractive, or the way in which trade-offs between conflicting outcomes are negotiated. Thus, whereas the literature suggests that fidelity and adaptation decisions can be justified based on how different outcomes are valued, there is a knowledge gap concerning how this appraisal process plays out in decision-making. This is particularly important, considering that decisions related to fidelity and adaptation represent choices between options that may have different configurations of values—some that make them attractive and some that do not. Knowledge of how outcomes are appraised is therefore essential for understanding how decisions related to fidelity and adaptations are made.

### Making decisions about fidelity and adaptation

Although decisions affecting fidelity and adaptation can be made not only at the individual but also at the organizational and system levels, frontline practitioners (e.g., service providers in health or social care organizations) sit at the nexus of the fidelity–adaptation dilemma. They (1) tend to have the most in-depth understanding of the local service context and the fit of an EBI, (2) are the direct targets of EBI training and professional development strategies, and (3) are the default (and frequently unsupported) fidelity–adaptation decision-makers. Indeed, for EBI practitioners, the fidelity–adaptation dilemma is not a philosophical or theoretical question. It entails a complicated decision process wherein practitioners need to weigh their options for action based on several—sometimes conflicting—outcomes. The way in which these decisions are made remains largely unexplored in the implementation literature and practice.

A decision is formally defined as “a commitment to a course of action that is intended to yield results that are satisfying for specified individuals” [26]. Two theories, expected utility and ecological rationality, provide somewhat different perspectives on decision-making. The expected utility theory is a “rational theory” proposing that people act to maximize utility—that is, they act based on a holistic estimate of the total value, or satisfaction, that a choice will offer [20]. Thus, utility is frequently used to operationalize value as a multicomponent, multilevel construct [20]. The theory of expected utility describes how people make decisions according to structured processes wherein rules of logic or probability are applied to optimize the utility of a decision [27]. This includes a series of steps, starting with identifying a problem that calls for a decision and the available options for action, followed by an assessment of the possibilities and risks of each option and the anticipated impact on outcomes [28]. In contrast, ecological rationality theory is a bounded rationality theory that describes how decisions are influenced by the task at hand, individual factors, and the environment in which they are made [29, 30].

The literature on EBI fidelity and adaptations suggests that decisions are optimized when made through a structured process, proactively and with careful consideration of how they will affect core components of an EBI and, ultimately, its effectiveness [19, 31–34]. This reflects a rational approach to decision-making [35] that mimics the decision-making process described by expected utility theory. However, practitioners often make decisions about fidelity or adaptations under bounded conditions that are more in line with ecological rationality—conditions under which a lack of time and think-space leads to ad hoc and implicit decisions with limited consideration of consequences [24, 29, 36]. Thus, theoretically, the appraisal of which outcomes are most valuable is central in decisions related to the fidelity–adaptation dilemma [27]. This is particularly true when the outcomes are in conflict, which means that the decision involves a trade-off between outcomes. Nevertheless, the validity of these theoretical perspectives in relation to the fidelity–adaptation dilemma has yet to be investigated.

The lack of knowledge of how outcomes are valued and how this affects fidelity–adaptation decisions limits our understanding of which outcomes EBIs should be valued against to optimize their benefit. This calls for studies on the relationship between value appraisals and decisions to adhere or adapt.

### Aim and research questions

Inspired by decision-making theory, the objective of this project is to explore how practitioners appraise the values of the outcomes of EBI implementation and to test how this appraisal influences decisions related to fidelity–adaptation dilemmas.

The project consists of three sub-studies. Sub-study 1 is exploratory and aims to uncover the dilemmas, options, valued outcomes, and value trade-offs in fidelity and adaptation decisions (research questions [RQ] 1–4). This will inform Sub-study 2, a discrete choice experiment that will test the impact of value configurations on decisions related to fidelity and adaptation (RQ5). The explorative and experimental findings of Sub-studies 1 and 2 will form the basis of Sub-study 3, a mixed-method study aiming to shed light on the differences between optimal and constrained decision situations (RQ6).

#### Sub-study 1: exploring fidelity–adaptation parameters

RQ1. What are the typical fidelity–adaptation dilemmas that practitioners encounter?

RQ2. What options for action are there for practitioners facing these typical dilemmas?

RQ3. What outcomes are valued by the various stakeholders in decisions related to fidelity–adaptation dilemmas?

- What are the value appraisals associated with different options for practitioners?
- How do value appraisals differ between stakeholders (i.e., service users, practitioners, and decision-makers)?

RQ4. What value conflicts do practitioners encounter, and how are trade-offs made?

#### Sub-study 2: experimentally testing rational fidelity–adaptation decision-making

RQ5. How do experimentally manipulated value configurations explain choices between fidelity and adaptation?

- Which option do practitioners prefer?
- How do value appraisals influence decisions?
- What trade-offs are practitioners willing to make to improve a certain value? What risks are they willing to take?

#### Sub-study 3: contrasting experimental and real-world value trade-offs

RQ6. How do practitioners make sense of decisions made in optimal decision situations (experiment) and constrained, real-world decision situations?

### Theoretical approach

This project uses decision-making theory to understand how value appraisals influence decisions related to the fidelity–adaptation dilemma. It combines the theories of expected utility and ecological rationality, which will allow us to understand how decisions about fidelity and adaptation are made rationally, in line with current recommendations in implementation research [19, 31–34], as well as how decisions are often made in practice, under bounded conditions.

Expected utility theory is specifically used to outline the parameters explored (Sub-study 1) and manipulated in the experiment (Sub-study 2). To understand decisions, the first step is to identify the *options* (RQ2) available in typical fidelity–adaptation dilemmas (RQ1). The next step is to understand how the potential outcomes of these options are valued—that is, how the different options are judged based on the configuration of outcomes (RQ3). Theoretically, each outcome, called an *attribute*, can be conceptualized on different *levels*, such as being more or less efficient or user-centered. After identifying options, attributes, and levels, we will explore the decision situation as a whole, including value conflicts and trade-offs, such as when one option maximizes effectiveness but reduces reach and another increases reach but sacrifices effectiveness (RQ4).

The theory of expected utility is a normative theory in that it describes how people *should* make decisions in light of uncertainties, such as when practitioners need to choose between options that all include some kind of risky prospects or the fidelity–adaptation dilemma [27]. However, in many decision situations, there are constraints that limit time and information, and the “more-is-more” approach suggested by expected utility theory may not always be feasible [27]. Bounded rationality is a broad theoretical framework used to describe how people, making the most of their cognitive ability in a complex environment that restricts the use of comprehensive decision-making processes, tend to focus on finding good enough solutions. For example, they stop searching for options as soon as they identify one that exceeds a certain aspiration level or meets certain criteria [30]. The shortcomings and risks associated with applying heuristics have received significant interest (e.g., by Nobel Prize laureate Daniel Kahneman), including with regard to clinical decision-making [37]. However, it has also been pointed out that the use of heuristics may not mean that people are irrational but rather ecologically rational—that is, adaptive in that they use a “less-is-more” approach to making decisions in contexts that are known to them [29]. Ecological rationality theory and its perspective on bounded rationality will inform the contrasts explored in RQ6. In this, we will follow recommendations to compare the experimental findings with real-world data [38].

## Methods

### Design

**Fig. 1:**
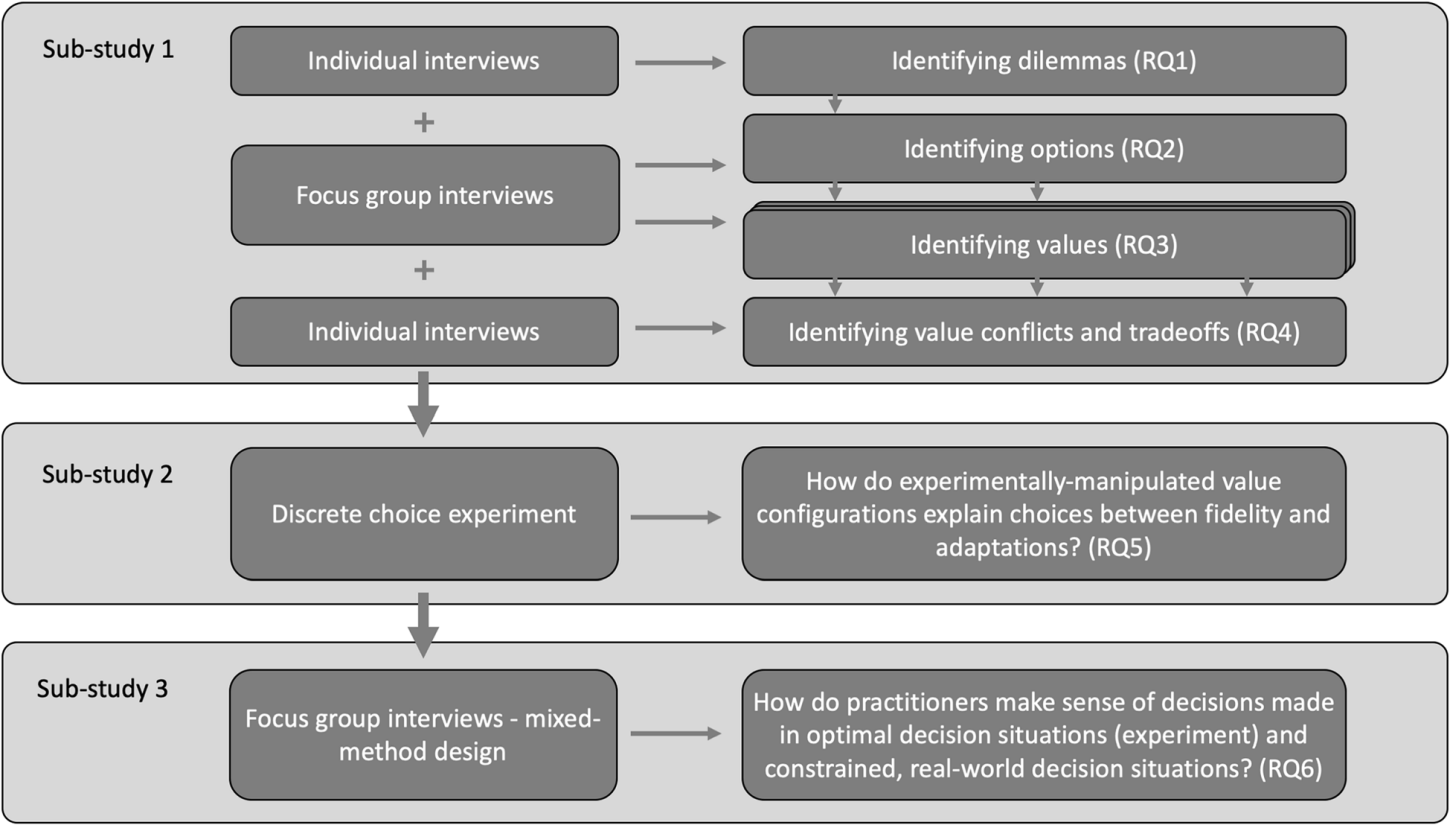
Outline of study design and research questions

This is a multi-method study consisting of three sub-studies: a qualitative, exploratory study (Sub-study 1), a discrete choice experiment conducted through a survey (Sub-study 2), and a mixed-method study (Sub-study 3) (Fig. 1). The findings will be reported using the Good Reporting of A Mixed Methods Study guidelines [39].

### Setting

This is a nationwide study set in Sweden that will focus on parental programs as the target EBIs. Parental programs are psychosocial interventions that aim to improve parenting practices and behaviors, covering the prevention–intervention spectrum (i.e., from universal to indicated prevention programs) and often delivered in community contexts. Parental programs are well suited for studying fidelity–adaptation decision-making because there are multiple programs available that (1) have strong empirical support [40], (2) contain established fidelity assessment rubrics, (3) have broad uptake in practice settings, and (4) involve several categories of professionals. Applying these criteria to the Swedish context, we identified six programs for likely inclusion: All Children in Focus [41], Comet [42], Cope [43], Incredible Years [44], Triple-P [45], and Connect [46]. Parental programs are also on the policy agenda in Sweden, being promoted by the national government, and thus have broad policy and practical implications.

### Participants and recruitment

In Sweden, parental programs are primarily the responsibility of local and regional government agencies. Consequently, there are 290 municipalities and 21 regions that can potentially participate. Within these organizations, the primary participant group will be practitioners implementing evidence-based parental programs, including several categories of professionals (e.g., social workers, preschool teachers, psychologists, and nurses). Additionally, service recipients and decision-makers (e.g., managers and policy makers) will be recruited to address RQ3.

The participants will be independently invited to each sub-study, although it is anticipated that some may participate in more than one (e.g., focus groups and experiment). For Sub-studies 1 and 3, we will use stratified purposeful sampling [47] to ensure the representation of municipalities of all sizes and both rural and urban areas. Organizations will be invited to participate based on the information provided on their websites. There is also an established collaboration supporting reach-out with the Family Law and Parental Support Authority, which plays a national coordinating role in the implementation of parental programs in Sweden.

If the initial contact with an agency is positive, all professionals working on parental programs in that organization who have formal training in a parental program that meets the abovementioned criteria and have practical experience in leading parental groups will be invited to participate in the study. RQ3b also targets service users (parents) and decision-makers (managers and policy makers). These will be recruited through the participating practitioners using a snowball sampling approach. In Sub-study 2, we will invite all potential participants identified in the previous steps.

### Sub-study 1: data collection and analysis

A combination of qualitative methods will be used to address RQ1–4 and to ensure the external validity of the experiment (Sub-study 2) by basing it on empirical data [20]. First, fidelity–adaptation dilemmas (RQ1) will be explored through about 10 individual interviews with parental program practitioners, aimed at understanding how practitioners describe typical fidelity–adaption dilemmas. Throughout the study, the numbers of interviews will depend on the richness and complexity of the data [48]. This means that the numbers of respondents cannot be determined beforehand.

Second, sequential focus group interviews [49] with 4–8 participants in each group will be conducted to explore the alternatives between which practitioners choose (i.e., options [RQ2]) and the outcomes that influence their choices (i.e., attributes [RQ3a]). Inspired by the procedure of Coast and Horroks for exploring attributes [50], we will use an iterative approach whereby data collection and analysis proceed concurrently. Once the analysis indicates that options have been thoroughly explored, we will gradually shift the emphasis from primarily investigating options in the initial focus groups to the outcomes that are valued with those options (i.e., the attributes). This shift also entails moving from a fully explorative approach in the first focus group, in which no option has yet been identified, to a sequential interview format, in which the exploration of options is followed by the presentation of the options identified in previous focus groups. This approach allows the exploration of the anticipated outcomes of all options and the way in which they are valued. With this iterative approach, there is no fixed set of focus groups planned in advance. Based on previous research, we anticipate three to four practitioner focus groups [51]. Another two to three focus groups of parents and decision-makers, respectively, will be formed to explore how these stakeholders appraise the value of parental groups (RQ3b).

Third, another round of *individual interviews* with practitioners (RQ4) will be conducted. Previously collected data will be used as input to elicit information about the value conflicts and the associated trade-offs that practitioners experience. The interviews will include two phases, inspired by Grundstein et al.’s study on ethical decision-making [52]. First, the participants will be asked to describe critical incidents in which they had to make a decision related to the fidelity–adaptation dilemma and the value trade-offs involved. Second, the dilemmas and associated options and valued outcomes identified in the previous steps will be presented, and value conflicts will be explored. In this, we will also collect data on how each outcome varies, which will subsequently inform the selection of response categories (i.e., levels) for the attributes when constructing the experiment (RQ5). Data collection, transcription, and analysis will be conducted iteratively. We estimate that 15–20 interviews will be required.

#### Data analysis

Data from all qualitative methods in Sub-study 1 will be analyzed using reflexive thematic analysis [53, 54]*.* The interviews will be recorded and transcribed verbatim. Two persons will conduct the analysis and reflectively compare each other’s interpretations of the data. To evaluate the study’s trustworthiness, Guba and Lincoln’s [55] criteria and recommendations for verification strategies will be used. The remaining researchers will act as informed outsiders.

### Sub-study 2: data collection and analysis

After exploring the parameters underlying decisions about fidelity and adaptation, to address RQ5, we will conduct a discrete choice experiment using an online survey to test how different combinations of attributes and levels affect choices [20].

The experiment will mimic real-world decisions. Practitioners will make decisions based on a holistic appraisal of two options—adhering or adapting. The options differ in terms of combinations of attribute levels—that is, they offer some outcomes with more value and others with less (i.e., different configurations of values). The value configuration will serve as the independent variable, and the choice between adhering or adapting will be the dependent variable. In the hypothetical example shown in Table 1, the attributes (outcomes) are efficacy, timeliness, and two types of adverse outcomes—one related to those receiving the EBI and one related to those not receiving it, and the dependent variable is adherence to or adaptation of the program.

**Table 1:** A hypothetical example of a survey question in the discrete choice experiment

Scenario: The parental program includes 10 sessions, seven focusing on improving parental skills in general and parent–child relationships in particular, and three focusing directly on child behavioral problems. Each group includes six parents, and due to constraint resources, the practitioners can only accommodate 21 sessions per year. There is a long waiting list.		
**Outcomes**	**Option 1: Provide all 10 sessions**	**Option 2: Omit three sessions**
Efficacy	Parental skills are improved, and relational and behavioral issues are solved.	Parental skills are improved, and relational issues are solved, but behavioral problems remain.
Timeliness	Twelve parents a year are treated.	Eighteen parents a year are treated.
Adverse outcome, participants	One chance in 20 that behavioral problems will deteriorate to delinquency.	One chance in 10 that behavioral problems will deteriorate to delinquency.
Adverse outcome, waiting list	One chance in 10 that children on the waiting list develop anxiety disorder.	One chance in 20 that children on the waiting list will develop anxiety disorder.
**Which option would you choose?**	**Option 1**	**Option 2**

#### Experimental survey development

Scenarios (i.e., typical dilemmas; RQ1), options (RQ2), attributes (RQ3), and the response categories that are relevant to an attribute (i.e., levels; RQ4) will be identified through the preceding qualitative steps (Sub-study 1). A central part of the experiment consists of the determination of the number of scenarios and questions in the survey. The number of possible combinations of attributes and levels increases exponentially (e.g., five attributes with five levels each equals 3125 combinations). Therefore, we will use a fractional factorial experimental design. This involves reducing the number of combinations statistically [56].

If the pre-work indicates that some value judgments do not vary meaningfully, we may opt for a partial profile design, in which the level of specific attributes is held constant [57]. This will also be done if there are more than five to six outcomes associated with a specific scenario to reduce the cognitive load associated with judging a large number of attributes [57] and to minimize the risk of the survey being perceived as too time-consuming and/or difficult [58].

The experiment will include approximately four scenarios, each with a number of questions where the respondent will be asked to choose between two or more options (i.e., *option-choice questions*). We will aim for around 96 option-choice questions, 24 for each dilemma. Each respondent will be randomly assigned to one of six versions, meaning that each participant will respond to 16 questions, four per scenario. Up to 16 questions are commonly used as a trade-off between cognitive load and data acquisition [59]. The sequence of scenarios will be randomized. For each question, participants will be asked to indicate how certain they are about their choice, to assess choice certainty [60]. In addition, the stability and rationality of responses will be tested. Choice consistency (if the same choice is made twice) will be tested by repeating one question in each survey, and rationality by including a discrete choice comparison where one alternative is superior to the other (choice monotonicity) [60]. Data collected through the survey will also include demographic information, education, professional experience, and experience in evidence-based parental programs.

#### Data collection

After careful pilot testing, participants will be sent emails with a link to a secure web-based survey. The required sample size for an experiment depends on the numbers of choice tasks, alternatives, and analysis cells [59]. A rule of thumb is that a sample size of over 100, or 20 respondents per questionnaire version (six in this case), is required [59]. Based on this, we aim for at least 120 respondents, which we deem feasible given that, according to a conservative estimate, at least 1000 practitioners work on parental programs in Sweden.

#### Data analysis

The data will be analyzed using random parameters logit or multinomial logit models, depending on whether the scenario includes a choice between two or more than two options. Nested models may be an alternative if they better reflect how decisions are made (i.e., if decisions follow a decision-tree structure). We will use effect coding rather than dummy coding, as this allows the estimation of effect sizes for each attribute level. Individual utility profiles will be modeled, from which we will estimate parameters reflecting which option practitioners prefer, including the magnitude, sign, and statistical significance of coefficients; the relative importance of different outcomes; how the configurations of values influence choices; how changes in value configurations affect the probability of a certain decision; and willingness to trade—that is, what a person is willing to give up in one outcome to improve another (the marginal rate of substitution) [56].

Differences in decisions between stakeholders and practitioners with different levels of experience will be analyzed through split-sample analysis using Log likelihood Chow tests to examine whether preferences differ between subgroups.

To determine trade-off levels, we will calculate the maximum acceptable risk (from preference weights)—that is, the greatest risk that participants are willing to accept for a given outcome or the marginal rate of substitution. The experiment will thus provide information on which decision, given the known value configurations, represents the optimal balance between the different outcomes.

### Sub-study 3: data collection and analysis

Sub-study 3 will use a mixed-method design [61]. We will conduct three to four focus group interviews using the findings of Sub-studies 1 and 2 as input, thus inviting the participants to elaborate and expand on the findings from the experiment, which represent a highly structured decision situation, and interviews, which provide information about less ordered, constrained decision situations. We will contrast qualitative (RQ1–4) and quantitative (RQ5) data and then collect additional qualitative data to address a new research question (RQ6) [62]. An interview guide will be developed based on the dilemmas and value conflicts identified. For each dilemma, the respondents will be shown graphical summaries of findings from the interviews and the experiment. The interview guide will be semi-structured around each dilemma and will point practitioners to value conflicts. However, the questions will be open-ended, for example, “What are your thoughts on the results?” and “How does this reflect your own experience?”

#### Data analysis

The data will be analyzed using the six-step reflexive thematic approach, which aims to identify themes and make sense of patterns of meanings in the data [53, 54].

## Discussion

This study is unique in its attempt to approach the fidelity–adaptation dilemma in the context of practitioners’ decision-making processes. Decision theories are used to first explore and then test how practitioners appraise the values of different outcomes of EBI implementations and how this value appraisal influences decisions related to fidelity–adaptation dilemmas. This decision-making perspective will make a significant contribution to the literature, which to date has primarily either provided general recommendations on how decisions should be made or described when, what, how, and by whom (but seldom why) adaptations are made.

Furthermore, the project will provide an example of how discrete choice experiments can be used to understand decisions affecting fidelity and adaptations when practitioners use EBIs. Discrete choice experiments have been used extensively in health economics, for example, to evaluate patient experiences and health outcomes and assess trade-offs in outcomes and clinical decision-making [63]. It has been suggested that discrete choice experiments can be used as a stakeholder engagement strategy to identify attributes of EBIs and implementation strategies that can make the adoption of an EBI more attractive [64–66]. For example, it can be used to select and tailor implementation strategies to the needs of providers [67] or to barriers and facilitators [58] and to evaluate the appropriateness of these strategies [68]. Its advantages over traditional surveys include a more granular understanding of people’s decisions based on how they value options [67] and a better reflection of the trade-offs often involved in decisions related to implementation [58]. Despite its suggested benefits [64, 67], the use of discrete choice experiments in implementation research is still in its infancy and has, to our knowledge, not yet been used to explore the fidelity–adaptation dilemma and the influence of value configurations on practitioners’ decisions.

This study is also original in terms of its focus on how different outcomes are valued and drive decisions. Debates about the fidelity–adaptation dilemma in the literature have primarily focused on how it affects effectiveness, whereas practitioners are obligated to consider multiple types of outcomes in their decision-making. Thus, this project will offer a rare insight into how considerations of the values of different outcomes affect practitioners’ decisions related to fidelity and adaptations. Such knowledge is necessary for producing solid recommendations on how fidelity and adaptation decisions should be made. Thus, the project will provide a deeper understanding of how the values of EBI outcomes are appraised and how implementation success is to be determined.

## Data Availability

The datasets used will be available from the corresponding author on reasonable request.

## Abbreviations

EBI: Evidenced-based intervention
